# Motivation or involution: a study on the causes and coping strategies of the occupational stress of young teachers in universities

**DOI:** 10.3389/fpsyg.2026.1831517

**Published:** 2026-05-15

**Authors:** Jiajia Wei, Xinglong Xu, Sani Mahmud Affa

**Affiliations:** Jiangsu University, Zhenjiang, China

**Keywords:** colleges and universities, young teachers, occupational stress, strategies, Zhenjiang city

## Abstract

**Introduction:**

Amid ongoing reforms in higher education, young university faculty are experiencing mounting occupational stress. This study investigates the stress levels and contributing factors affecting young teachers in nine universities in Zhenjiang, Jiangsu Province.

**Methods:**

Data were collected via a questionnaire survey using convenience sampling, yielding 300 valid responses. Through descriptive statistics, correlation analysis, and regression modeling, the study examines key dimensions such as career development, guarantee for daily life, interpersonal relationship, role responsibilities and self-expectations, organizational and management, assessment system.

**Results:**

Factors such as age, daily working hours, income, career development opportunities, and organizational climate significantly impact stress levels.

**Discussion:**

Based on the findings, the paper proposes targeted strategies to alleviate stress, including increasing faculty autonomy, fostering collaboration, improving research environments, and promoting a positive mindset.

## 1 Introduction

The report delivered at the 20th National Congress of the Communist Party of China emphasized that “education, science and technology, and human resources are the foundational and strategic pillars for building a modern socialist country in all respects.” The report also called for adhering to the principle of prioritizing educational development, delivering education that meets the expectations of the people, fully implementing the Party’s educational policies, and accelerating the development of a high-quality education system. Over the years, the Party and the state have consistently recognized education and human resources as fundamental strategies for national development. In this context, young university faculty members have emerged as the vanguard of educational progress, playing crucial roles in talent cultivation, advancing scientific research, and contributing significantly to the realization of the great rejuvenation of the Chinese nation.

Nevertheless, the expansion of higher education enrollment has intensified the reform of the sector. In pursuit of institutional competitiveness, many universities have introduced performance-driven policies such as “up or out,” “competitive recruitment,” and “last-place elimination.” While these policies may create development opportunities for young faculty, they simultaneously impose substantial pressure. Young university teachers, who are often at the frontlines of teaching and research, are burdened with increasingly heavy workloads. Persistent stress, if left unaddressed, can result in adverse outcomes ranging from loss of motivation to deteriorating physical and mental health.

Extensive research has been conducted both internationally and domestically to understand the multidimensional nature of occupational stress. There are four main tools for measuring work stress in foreign countries: Cooper’s Occupational Stress Inventory (OSI) and McLean’s Work Stress Scale, which are based on the theory of the “Person-Environment Fit Model”; and Karasek’s Job Content Scale and Hurrell’s Job Control Scale, which are based on the “Job Demand-Control Model.” Commonly used scales for measuring teacher stress include the Professional Life Stress Scale (PLSS) developed by Fontana (1989), the Teaching Events Stress Inventory (TESS) developed by Cichon and Koff (1980), the Teaching Occupational Stress Factor Questionnaire (TOSFQ) developed by Clark (1980), and the Maslach Burnout Inventory (MBI) and Burnout Measure (BM) by Maslach and Pines.

Some domestic researchers directly use foreign work stress scales, such as Lin et al. (2003) who used the questionnaire on work stress and work stress response prepared by the University of South Florida to measure the work stress of university staff (Shi, 2005); Some researchers revised the foreign scale to localize it, such as Wang et al. (2003) and others revised a teacher burnout scale suitable for Chinese culture (Wang et al., 2003); Some of the self-made scales, Li (2005) self-made China University Teacher Stress Scale, systematically measured the type and intensity of domestic university teacher stress. Subsequent studies such as (Zeng, 2010; Lin and Wu, 2017; Zhang et al., 2022) have used the scale, and repeatedly verified that it has good reliability and validity, and has a mature application foundation. At present, there is still a lack of widely recognized assessment tools for the unique stress of university teachers in the domestic academic community. The scale has been recognized in the field, so this paper will use it for reference to carry out work stress measurement.

This study aims to investigate the current state of occupational stress experienced by young faculty members at nine universities in Jiangsu Province. Drawing upon the China University Teacher Stress Scale developed by Li (2005), this study focuses on six core dimensions: career development, Guarantee for daily life, interpersonal relationships, Role responsibilities and self- expectations, Organizational and management, and Assessment system. By examining the demographic profiles of respondents and these specific dimensions, this study explores the root causes of stress and offers targeted strategies to mitigate its effects. The goal is to provide practical recommendations that can help alleviate occupational stress and safeguard the well-being of young university educators.

## 2 Literature review

Occupational stress among young university teachers has been a growing concern in recent years, particularly as higher education institutions undergo structural and policy reforms. Recent studies have examined the psychological, organizational, and socio-economic factors contributing to stress and burnout among educators.

### 2.1 Occupational stress in higher education

Occupational stress among university faculty has gained significant scholarly attention globally, especially as higher education systems undergo profound changes. The Job Demands-Resources (JD-R) Model (Bakker and Demerouti, 2023) provides a foundational theoretical framework, positing that burnout and engagement are respectively caused by high job demands and abundant job resources. Mudrak et al. (2017) applied this model in European academic settings, demonstrating that psychological strain among faculty is significantly affected by the imbalance between teaching, research, and administrative duties. Naidoo-Chetty and du Plessis (2021) similarly found that administrative overload and the erosion of academic autonomy were major contributors to occupational stress among South African lecturers. The COVID-19 pandemic further complicated this dynamic. According to Demerouti and Bakker (2014), the transition to remote work and blurred work-life boundaries exacerbated burnout risk, especially among early-career faculty. Additionally, Kinman and Wray (2020) argue that institutional responses during the pandemic prioritized performance over wellbeing, neglecting the psycho-social needs of faculty. These studies collectively underline that academic stress is a function of systemic structures rather than solely personal resilience.

### 2.2 Neoliberalism, audit culture, and academic precarity

Academic institutions globally have increasingly adopted neoliberal management practices characterized by accountability, competition, and performativity. Gill (2014) critiques how academics are subjected to audit cultures frequent performance evaluations and quantifiable outputs which erode job satisfaction and amplify stress. Mountz et al. (2015) propose “slow scholarship” as a counter-response to such pressures, emphasizing time for reflection, collaboration, and intellectual depth. The situation is compounded by academic capitalism (Slaughter and Rhoades, 2019), where market logic penetrates research agendas, funding structures, and institutional priorities. In such environments, early-career scholars often face precarious employment and an overreliance on performance metrics for career advancement (Loveday, 2018). According to Burrows (2019), such performative cultures have normalized anxiety and burnout, particularly among those striving for tenure or promotion. These critiques are essential in understanding the structural sources of occupational stress, beyond individualized explanations.

### 2.3 The phenomenon of “involution” in Chinese academia

The concept of “involution” (neijuan) has become a potent analytical lens in the Chinese academic discourse. Originally used in anthropology, “involution” now describes the paradox of hyper-competition yielding diminishing returns. Wang et al. (2023) contextualizes this in academia, where excessive publishing, grant applications, and administrative tasks create a treadmill effect with little personal or professional payoff. Bao et al. (2021) argue that China’s massification of higher education has intensified involution, especially in urban research universities. While policies like “publish or perish” aim to foster excellence, they often lead to psychological exhaustion and disillusionment among young faculty. Ma (2025) emphasize that involution is exacerbated by limited career mobility and high housing costs in academic cities, making overwork a necessity rather than a choice. Zhang et al. (2024) provides empirical evidence showing that young scholars experience a sense of futility and existential stress due to this competitive culture. These insights are crucial for distinguishing the stress of young faculty in China from that experienced in other national contexts.

### 2.4 Psychological capital and coping mechanisms

Psychological capital (PsyCap), encompassing self-efficacy, optimism, hope, and resilience, is increasingly recognized as a key moderator of occupational stress. Luthans et al. (2021) argue that high levels of PsyCap enable employees to adapt better to stressors and maintain productivity. In the academic context, Shen and Guo (2025) found that Chinese university teachers with higher PsyCap reported lower burnout and higher teaching satisfaction. Gan and Cheng (2021) further demonstrate that psychological capital mediates the relationship between institutional support and well-being, highlighting the role of organizational environments. Interventions to build PsyCap such as mindfulness training, resilience workshops, and peer mentorship have been empirically linked to lower stress levels (Avey et al., 2011; Yin et al., 2025). However, some scholars caution against overemphasizing individual coping without addressing institutional causes. Misra and others (Griffin, 2021) argue that such approaches risk shifting responsibility from institutions to individuals, thereby masking deeper structural reforms.

### 2.5 Gender and intersectional stressors

Occupational stress is not monolithic; it intersects with gender, race, age, and family status. Griffin (2021) discusses the “ivory ceiling,” where female faculty disproportionately undertake service work, mentoring, and emotional labor. This invisible workload exacerbates stress and impedes research productivity. O’Meara et al. (2017) similarly find that women are more likely to experience role conflict and work-life imbalance, particularly in male-dominated departments. Su and Jiang (2023) note that female academics often face societal expectations related to marriage and childcare, which compound occupational demands. According to Wang and others (Li, 2025), lack of institutional support for parental leave and flexible work arrangements further disadvantages women, especially in early career stages. Intersectionality offers a framework to understand how overlapping identities shape stress experiences. Crenshaw (1989) emphasized that race and gender cannot be examined separately, a view increasingly applied in academic labor studies. Addressing these complexities requires not only individual resilience but also equity-oriented institutional policies.

## 3 Materials and methods

### 3.1 Participants

This study employed a convenience sampling method to conduct a questionnaire survey targeting young university faculty members under the age of 40 across nine universities in Zhenjiang, Jiangsu Province. This age threshold was selected based on the widely recognized academic and institutional definition of young faculty, who typically face the dual pressures of establishing their research careers and assuming heavy teaching responsibilities during this critical career stage. Scholars and university administrators commonly regard those under 40 as being in a key developmental period, during which they must rapidly accumulate research achievements, apply for research projects, and adapt to increasingly competitive academic evaluation systems. Moreover, this group often bears heavy family burdens while striving for professional promotion, making them particularly vulnerable to work related stress. A total of 320 questionnaires were distributed and successfully returned, yielding a 100% response rate. After data cleaning and screening, 300 valid responses were retained for analysis, resulting in an effective response rate of 93.75%. This study was conducted in accordance with the Helsinki Declaration. All participants provided informed consent forms prior to data collection. The questionnaire was anonymous and participants were assured that they have the right to withdraw at any time. No ethical issues were found during the research process.

### 3.2 Scale design

#### 3.2.1 Instrument

Based on the professional particularities of young teachers in Chinese colleges and universities, the Occupational Stress Scale for Young Teachers in Colleges and Universities developed in this paper draws on “Development and Reliability and Validity Indicators of the Work - Stress Scale for University Teachers” developed by Professor Li Hong from Tsinghua University (Li, 2005), along with its established development procedures and psychometric indicators. Although the original scale was developed in previous studies, it has been widely cited and applied in domestic research on university teachers’ occupational stress, with stable reliability and validity verified in multiple representative samples. To ensure its applicability to the current sample of young faculty under 40 in Jiangsu Province, a rigorous re-examination of psychometric properties was conducted in accordance with standard scale validation procedures.

The scale consists of 23 items across six dimensions: Career development (5 items), Guarantee for daily life (3 items), Interpersonal relationship (4 items), Role responsibilities and self-expectations (4 items), Organizational and management (4 items), and Assessment system (3 items). Responses are provided on a 5-point Likert scale (1 = “strongly disagree” to 5 = “strongly agree”). Both dimensional and total stress scores were computed as mean values. Higher mean scores reflect a higher intensity of perceived occupational stress, with no specific clinical cut-off points applied in this study. The final item on the scale “How do you perceive your overall stress level?” served as a direct indicator of respondents’ occupational stress. All items employed the same five-point Likert scale, with higher scores representing greater perceived stress.

In this study, the dependent variable is the overall level of perceived occupational stress, while the independent variables are the six stress dimensions listed above.

#### 3.2.2 Control variables

Control variables included participants’ demographic and professional characteristics: gender, age, marital status, education level, type of institution, professional title, years of experience, average daily working hours, annual income, and primary motivation for engaging in academic research.

### 3.3 Data analysis strategy

The data were analyzed using SPSS 23.0. The analysis process was organized into the following four stages.

#### 3.3.1 Reliability and validity of the instrument

##### 3.3.1.1 Validity test

To rigorously confirm the psychometric quality of the scale in this study, a sequential validation procedure was implemented. To test the construct validity of the scale, this study employed Exploratory Factor Analysis (EFA). Before factor extraction, two applicability tests are first conducted to determine whether the data is suitable for factor analysis: Kaiser Meyer Olkin (KMO) sampling appropriateness scale, which is used to test the partial correlation between variables. Its implicit assumption is that there is sufficient common variance between variables. According to Kaiser’s (1974) standard, a KMO value greater than 0.6 is an acceptable threshold; Bartlett’s sphericity test is used to test whether the correlation matrix between variables is an identity matrix (i.e., independent of each other). Its implicit assumption is that there is a significant correlation between variables, and this test needs to reach a statistically significant level (*p* < 0.05) to reject the null hypothesis (Bartlett, 1951). Factor extraction uses Principal Component Analysis (PCA), which assumes that the common variance between variables can be explained by a few linear combinations. The number of factors is determined based on eigenvalues greater than 1 (Braeken and van Assen, 2017) and combined with the comprehensive judgment of steep slope points in the gravel map. Since it is assumed that there is no strong correlation between the extracted factors, the maximum variance method (Varimax) is chosen for orthogonal rotation to simplify the factor structure and enhance interpretability (Castro et al., 2015). The implicit assumption of using cumulative variance contribution rate as the core validity indicator is that the extracted common factors should be able to explain a sufficient proportion of the total variation of the original variables. According to Hair et al. (2019), the cumulative variance contribution rate should reach 60% or more.

##### 3.3.1.2 Reliability test

To evaluate the internal consistency of the items, the Cronbach’s alpha coefficient is used for validation. When the Cronbach’s alpha coefficient of the total questionnaire is greater than 0.7, for each dimension. If Cronbach’s alpha coefficient is greater than 0.6, it indicates that the questionnaire has good reliability (Taber, 2017). At the same time, homogeneity testing was conducted based on the changes in Cronbach’s alpha coefficient after deleting the item. The deletion criteria were: the Cronbach’s alpha coefficient corresponding to the deleted item was greater than the total Cronbach’s alpha coefficient of the questionnaire (Raykov and Marcoulides, 2019).

#### 3.3.2 Descriptive analysis

Descriptive statistics were calculated to determine the average and standard deviation of each dimension, exploring the average level of stress. A higher mean indicates a greater perceived stress level, while the standard deviation reflects the dispersion of responses (Nick, 2007).

#### 3.3.3 Correlation analysis

The Pearson correlation coefficient was used to analyze, in order to test the strength and direction of the relationship between variables. This method was chosen based on the assumption of normal distribution of the data, providing the necessary statistical basis and rationality verification for the construction of OLS regression model. Correlation coefficients were interpreted according to Cohen’s (1988) guidelines: | r| < 0.1 = negligible, 0.1 ≤ | r| < 0.3 = weak, 0.3 ≤ | r| < 0.5 = moderate, | r| ≥ 0.5 = strong (Schober et al., 2018).

#### 3.3.4 Regression analysis

Finally, Ordinary Least Squares (OLS) regression was conducted to explore the impact of each dimension on the overall stress level. OLS regression was chosen due to its robustness under assumptions of linearity, independence of errors, homoscedasticity, and normality of residuals, all of which were verified prior to analysis. OLS allows for the interpretation of each dimension’s marginal contribution to the total stress score (Moutinho and Hutcheson, 2011).

## 4 Results

### 4.1 Characteristics of the sample

Of the 300 valid responses, 144 were from male faculty (48%) and 156 from female faculty (52%). Regarding age distribution, 16% (*n* = 48) were aged 30 or below, 54.3% (*n* = 163) were between 31 and 35, and 29.7% (*n* = 89) were between 36 and 40. The majority (62%) held doctoral degrees or above.

In terms of institutional background, most respondents were from comprehensive universities (66.3%), and the most common academic title was lecturer (44%). A significant proportion of respondents had 1–5 years of teaching experience (45.7%) and reported working an average of 7–9 h daily (41%). Additionally, 86.7% reported annual incomes between 90,000 and 160,000 RMB.

When asked about their motivations for engaging in research, most young faculty cited “title evaluation” as their primary purpose, followed by “improving professional competence.” Fewer respondents selected “personal interest,” suggesting a career-oriented rather than intrinsically motivated research culture.

### 4.2 Reliability and validity of the instrument

#### 4.2.1 Validity test results

The analysis results show that the data meets all the assumptions of factor analysis. The KMO value is 0.753 (>0.6), and Bartlett’s sphericity test reaches a significant level (*p* < 0.001). Indicating sufficient common variance among variables, the data is highly suitable for factor analysis. Using principal component analysis and maximum variance rotation, six factors were extracted based on the criterion of eigenvalues greater than 1. As shown in Table 1, the cumulative variance contribution rate of these six factors is 75.807%, which is much higher than the recommended standard of 60%, indicating that the extracted common factors have good explanatory power for the total variation of the original variables. Overall, the scale has good construct validity, and the extracted six dimensions can effectively represent the measured construct.

**TABLE 1:** Factor loadings and variance explanation rates for each dimension.

Dimension	Item	Factor loading	Variance explained ratio (%)
Career development	There are fewer opportunities for further education and learning	0.812	18.45
	Difficult to apply for the project	0.798	
	Unclear career development prospects	0.785	
	There is a conflict between work and self-improvement	0.771	
	Intense competition for job positions	0.754	
Guarantee for daily life	Low salary and benefits	0.845	12.67
	The effort and return are disproportionate	0.831	
	Concerns about housing security	0.819	
Interpersonal relationship	Concerns of distrust from superiors	0.826	14.23
	Pressure from interpersonal relationships	0.808	
	I feel that my superiors have high demands	0.794	
	I feel that my colleagues have poor quality	0.776	
Role responsibilities and self- expectations	Society has high expectations for university teachers	0.837	13.58
	Students have high expectations for teachers	0.821	
	Strictly demand of oneself	0.809	
	Work requires a great deal of responsibility	0.793	
Organizational and management	The management institution for teacher occupational stress is not sound	0.843	12.91
	There are too many complicated procedures in work	0.828	
	The emphasis on scientific research and teaching varies among schools	0.814	
	Unreasonable organizational structure and low efficiency in handling affairs	0.796	
Assessment system	Rewards lean toward scientific research	0.852	11.95
	Professional title assessment did not take into account the characteristics of different disciplines	0.839	
	High requirements for the professional evaluation and appointment of teachers	0.825	
KMO value	0.753		
Bartlett’s sphericity test	*P* < 0.01		
Cumulative variance explanation rate	75.80%		

#### 4.2.2 Reliability test results

As shown in Table 2, the overall Cronbach’s alpha coefficient of this scale is 0.901, and the Cronbach’s alpha coefficient of each dimension is between 0.720 and 0.878, all higher than 0.70, which proves that the scale has satisfactory internal consistency reliability. The homogeneity test results showed that after deleting the six dimensions separately, the Cronbach’s alpha coefficient of the total questionnaire did not increase, so all dimensions were retained.

**TABLE 2:** Results of the questionnaire reliability analysis.

Project	Number of entries	Cronbach’s α coefficient	Cronbach’s alpha coefficient after dimension deletion (total questionnaire)	Result
General questionnaire	23	0.901	–	–
Career development	5	0.720	0.887	Retain
Guarantee for daily life	3	0.752	0.889	Retain
Interpersonal relationship	4	0.878	0.883	Retain
Role responsibilities and self- expectations	4	0.780	0.885	Retain
Organizational and management	4	0.815	0.882	Retain
Assessment system	3	0.850	0.880	Retain

### 4.3 Descriptive analysis

In order to further quantify the distribution characteristics of variables, this study conducted descriptive statistical analysis on all variables involved. As shown in Table 3, the statistical indicators include mean value, Standard deviation, 25% percentile, 75% percentile.

**TABLE 3:** Variable assignment and descriptive statistics.

Variables	Question items and assignment	Mean value	Standard deviation	25% percentile	75% percentile	Interquartile range
Dependent variable						
Perceived level of stress	I always feel pressure in my daily work	3.900	0.684	3.439	4.361	0.922
Independent variable						
Career development	There are fewer opportunities for further education and learning	3.824	0.532	3.464	4.184	0.72
	Difficult to apply for the project	3.915	0.485	3.588	4.242	0.654
	Unclear career development prospects	3.742	0.518	3.392	4.092	0.7
	There is a conflict between work and self-improvement	3.615	0.496	3.280	3.950	0.67
	Intense competition for job positions	3.698	0.465	3.384	4.012	0.628
Guarantee for daily life	Low salary and benefits	3.892	0.345	3.659	4.125	0.466
	The effort and return are disproportionate	3.865	0.332	3.641	4.089	0.448
	Concerns about housing security	3.703	0.308	3.495	3.911	0.416
Interpersonal relationship	Concerns of distrust from superiors	3.245	0.412	2.967	3.523	0.556
	Pressure from interpersonal relationships	3.338	0.385	3.078	3.598	0.52
	I feel that my superiors have high demands	3.356	0.368	3.108	3.604	0.496
	I feel that my colleagues have poor quality	3.273	0.345	3.040	3.506	0.466
Role responsibilities and self- expectations	Society has high expectations for university teachers	3.985	0.225	3.833	4.137	0.304
	Students have high expectations for teachers	3.942	0.212	3.799	4.085	0.286
	Strictly demand of oneself	3.968	0.198	3.834	4.102	0.268
	Work requires a great deal of responsibility	3.857	0.185	3.732	3.982	0.25
Organizational and management	The management institution for teacher occupational stress is not sound	4.125	0.215	3.980	4.270	0.29
	There are too many complicated procedures in work	4.098	0.205	3.960	4.236	0.276
	The emphasis on scientific research and teaching varies among schools	4.045	0.195	3.913	4.177	0.264
	Unreasonable organizational structure and low efficiency in handling affairs	4.044	0.188	3.917	4.171	0.254
Assessment system	Rewards lean toward scientific research	3.935	0.265	3.756	4.114	0.358
	Professional title assessment did not take into account the characteristics of different disciplines	3.885	0.248	3.718	4.052	0.334
	High requirements for the professional evaluation and appointment of teachers	3.868	0.228	3.714	4.022	0.308
Control variables						
Gender	Male = 1, Female = 2	1.520	0.500	1.183	1.857	0.674
Age	30 years old and under = 1, 31–35 years old = 2, 36–40 years old = 3	2.137	0.663	1.690	2.584	0.894
Marital status	Unmarried = 1, Married = 2, Divorce = 3, Widowed = 4	1.890	0.313	1.679	2.101	0.422
Educational level	Undergraduate students and below = 1, Master’s students = 2, Ph.D. students and above = 3	2.620	0.486	2.292	2.948	0.656
Type of university	Liberal arts colleges = 1, Science and engineering colleges = 2, Comprehensive universities = 3, Medical colleges = 4	2.503	0.756	1.993	3.013	1.020
Professional title	Teaching assistant = 1, Lecturer = 2, Associate Professor = 3, Professor = 4	2.297	0.724	1.809	2.785	0.976
Years of service	1–5 years = 1, 6–10 years = 2, More than 10 years = 3	1.717	0.743	1.216	2.218	1.002
Average daily working hours	2–3 h = 1, 4–6 h = 2, 7–9 h = 3; 10 h or more = 4	2.873	0.828	2.315	3.431	1.116
Annual income	80000 yuan and below = 1, 90000–160000 yuan = 2, 170000–250000 yuan = 3, 260000 yuan and above = 4	2.057	0.375	1.804	2.310	0.506
Purpose of conducting scientific research	Improving professional level = 1, Title evaluation = 2, Promote teaching = 3, Increase income = 4, Personal interest = 5	2.260	0.991	1.592	2.928	1.336

### 4.4 Correlation analysis

As shown in Table 4, Pearson correlation coefficient shows that the six dimensions of pressure are significantly positively correlated with the overall perceived pressure level (*p* < 0.01). Among them, career development (*r* = 0.652) and organization management (*r* = 0.501) were strongly correlated.

**TABLE 4:** Correlation analysis of core variables.

Variables	Career development	Guarantee for daily life	Interpersonal relationship	Role responsibilities and self- expectations	Organizational and management	Assessment system	Perceived level of stress
Career development	1.000						
Guarantee for daily life	0.488***	1.000					
Interpersonal relationship	0.284***	0.257***	1.000				
Role responsibilities and self- expectations	0.319***	0.473***	0.333***	1.000			
Organizational and management	0.426***	0.709***	0.231***	0.475***	1.000		
Assessment system	0.351***	0.166***	0.555***	0.314***	0.344***	1.000	
Perceived level of stress	0.652***	0.446***	0.466***	0.367***	0.501***	0.436***	1.000

*** means significant at the 1% statistical level.

Interpersonal relationships (*r* = 0.466), guarantee for daily life (*r* = 0.446), assessment system (*r* = 0.436) role responsibilities and self expectations (*r* = 0.367) were moderately correlated. This shows that the pressure level will rise synchronously with the increase of the scores of these dimensions, but the correlation size is different, which shows that although all dimensions constitute the overall pressure, they represent different aspects of the pressure, rather than repeated measurements.

Although some correlations are weak (e.g., role responsibilities and self expectations, *r* = 0.367), they still have statistical significance (*p* < 0.01). This is partly due to the relatively large sample size (*N* = 300), which improves statistical power and allows for the detection of smaller effect sizes. From a practical perspective, weak correlations indicate limited common variance between these dimensions and overall stress levels, which means they capture different aspects of occupational stress rather than redundant information.

### 4.5 Regression analysis

Conducting OLS regression analysis on each variable. Table 5 shows the results of the full sample regression analysis. Among them, Model 1 examines the relationship between the control variables and the dependent variable. Based on Model 1, Model 2, Model 3, Model 4, Model 5, Model 6 and Model 7 successively incorporate six dimensions, namely career development, guarantee for daily life, interpersonal relationship, role responsibilities and self-expectations, organizational and management, assessment system, to examine the influence of these six dimensions on the overall stress level of young teachers. This study also conducts a multicollinearity test on each variable.

**TABLE 5:** Results of the regression analysis of the full sample.

Variables	Model 1	Model 2	Model 3	Model 4	Model 5	Model 6	Model 7
Gender	−0.211***	−0.079	−0.079	0.085	0.074	−0.073***	0.055
Age	0.429***	0.282***	0.284***	0.173***	0.175***	0.137**	0.144**
Marital status	−0.232**	−0.169*	−0.169**	−0.116	−0.112	−0.099	−0.095
Educational level	0.080	−0.087	0.074	−0.048*	0.047	−0.034	−0.027
Type of university	0.063	0.082**	0.089**	0.107***	0.108***	0.107***	0.111***
Professional title	0.070	0.150**	0.124*	0.111*	0.105*	0.097	0.096
Years of service	−0.185**	−0.180***	−0.164***	−0.095	−0.095	−0.066	−0.072
Average daily working hours	0.206***	0.119***	0.116***	0.181***	0.178***	0.187***	0.184***
Annual income	−0.412***	−0.194**	−0.156*	−0.155**	−0.168**	−0.184**	−0.184**
Purpose of conducting scientific research	0.122***	0.107***	0.096***	0.100***	0.106***	0.087***	0.082***
Career development		0.546***	0.499***	0.456***	0.451***	0.436***	0.422***
Guarantee for daily life			0.335**	0.076	0.052	−0.045	−0.024
Interpersonal relationship				0.248***	0.236***	0.244***	0.218***
Role responsibilities and self- expectations					0.066	0.031	0.026
Organizational and management						0.186***	0.165**
Assessment system							0.061
R^2^	0.313	0.557	0.565	0.628	0.630	0.642	0.643
Adjusted R^2^	0.289	0.540	0.547	0.611	0.611	0.623	0.623
F	13.173	32.912	31.123	37.090	34.611	33.890	31.873

***, **, and * indicate differences at the statistical levels of 1%, 5%, and 10% respectively.

## 5 Discussion

### 5.1 The current situation of occupational stress of young teachers in universities

The findings indicate that occupational stress levels among young university teachers are generally high, consistent with earlier research by Ren and Zhang (2017). As shown in Table 1, the overall perceived stress score is 3.900 well above the neutral midpoint of 3 on the Likert scale. Additionally, the mean scores for all six dimensions of stress career development, life security, interpersonal relationships, role responsibilities and self-expectations, organizational management, and assessment and employment exceed 3.0, further confirming that stress among this demographic is widespread and multi-faceted.

This elevated stress may be largely attributed to recent institutional reforms and academic policies such as “publish or perish,” “competitive recruitment,” and “last-place elimination.” These policy environments, while intended to promote excellence, also intensify the psychological burden on young educators. In this study, a large proportion of respondents are aged between 31 and 35, have been working for 1–5 years, and typically work 7–9 h per day. This group represents an early-career cohort still navigating professional growth, career planning, and personal responsibilities such as housing and family support. These intersecting pressures create an environment of sustained occupational stress.

Furthermore, the survey revealed that the primary motivation for scientific research among respondents is professional title evaluation, with comparatively fewer respondents citing personal interest. This reflects a performance-driven academic culture in which research is often a means to meet institutional benchmarks rather than a pursuit of personal intellectual curiosity. As a result, stress levels tend to rise as faculty members strive to meet external expectations while sacrificing intrinsic motivation.

### 5.2 Analysis of the causes of occupational stress among young teachers in universities

Regression analyses identified several core stressors: age, average daily working hours, annual income, career development, interpersonal relationships, and organizational management. These variables significantly influence the occupational stress levels of young university faculty.

#### 5.2.1 Age

As shown in Table 3, age is positively correlated with stress. Older young faculty (particularly those in their late 30s) report higher stress levels. This aligns with findings by Zhang (2020). One possible explanation is peer competition and comparison. As faculty age, they may observe their peers achieving significant academic or professional milestones, which can generate internal pressure and self-doubt. Additionally, institutions often impose increasingly stringent criteria for promotion and evaluation as faculty members grow older, further amplifying stress.

#### 5.2.2 Average daily working hours

The results also demonstrate a significant positive relationship between longer working hours and higher stress levels. Despite the common perception of academia offering flexible schedules, many young teachers report long working hours due to both intrinsic and extrinsic factors. On the one hand, high self-expectations drive young faculty to strive for excellence in teaching and research. When outcomes fall short of these expectations, stress intensifies. On the other hand, institutional demands such as participation in administrative work, research projects, and student advising can dramatically increase workload. This accumulation of responsibilities often leads to fatigue and emotional burnout.

#### 5.2.3 Annual income

Annual income is negatively correlated with occupational stress. Faculty members earning lower salaries are more likely to experience financial stress, especially given the rising costs of housing, childcare, elder care, and other essential expenses. In cities with high living costs, young faculty often carry significant housing loans or car payments. Limited income, combined with high living expenses and family obligations, can contribute to long-term financial strain and associated psychological stress.

#### 5.2.4 Career development

Career development is one of the most significant predictors of occupational stress. Young faculty often encounter limited opportunities for further education, professional training, and project funding. Their relative lack of experience and social capital makes it difficult to access research resources or secure mentorship. In addition, unclear promotion pathways, insufficient institutional guidance, and the competitive nature of academic advancement all contribute to feelings of professional uncertainty and pressure.

#### 5.2.5 Interpersonal relationships

Interpersonal challenges also emerge as key stressors. Young faculty often report difficulties in navigating workplace dynamics with both superiors and colleagues. Some feel distrusted or undervalued by senior administrators, while others struggle with peer competition or group integration. These interpersonal tensions can create a hostile work environment, reduce collaboration, and increase social isolation, ultimately exacerbating stress.

#### 5.2.6 Organizational management

Organizational management received the highest average score among the six stress dimensions (4.078), indicating systemic institutional issues. Young faculty frequently express frustration with inefficient bureaucratic processes, excessive administrative burdens, and a perceived lack of institutional support. Many universities lack mechanisms for addressing faculty stress, and few provide accessible mental health resources or stress management programs. Without adequate structural support, young faculty are left to manage their stress independently, which may result in negative outcomes for both individual well-being and institutional performance.

## 6 Conclusions and recommendations

### 6.1 Summary of key findings

Young faculty members play an important role in the development of higher education in China. With their energy, innovation, and commitment to teaching and research, they are central to talent cultivation and academic advancement. While a moderate level of occupational stress may motivate professional growth and drive innovation, excessive stress can lead to diminished productivity, burnout, and adverse effects on physical and mental health (Li and Bao, 2017).

This study confirms that occupational stress is widespread among young university faculty and is significantly influenced by factors such as career development, interpersonal relationships, and organizational management. Additionally, age, income, and working hours serve as critical demographic and contextual contributors to stress. Therefore, mitigating stress requires a comprehensive and multi-level approach that involves individuals, institutions, and policy-making bodies.

### 6.2 Strategies for stress mitigation

#### 6.2.1 Empowering young faculty members

Young teachers in colleges and universities, due to their lack of seniority, have relatively limited access to effective resources. They have less-say within the organization. Even if they can put forward their own opinions, the likelihood of these opinions being adopted is relatively low. Young teachers in colleges and universities are a group full of responsibility and a sense of mission (Gmelch, 1993). They are eager to participate in school management and offer suggestions for the development of the school. Young teachers in colleges and universities are an important force in the school. Empowering young teachers with more power is conducive to the development of their creativity and the stimulation of their innovative thinking. It also plays a crucial role in relieving the occupational pressure of young teachers.

#### 6.2.2 Encouraging collaboration

Not only should mutual cooperation among young teachers within an organization be encouraged, but also the cooperation between young teachers from different colleges and universities should be promoted. Some young teachers in colleges and universities face relatively high occupational pressure due to improper handling of interpersonal relationships. Therefore, with the focus on the scientific research tasks that young teachers are concerned about, it is advisable to encourage them to cooperate with each other in scientific research, so as to achieve a win-win situation. In this way, it can not only make up for the deficiencies of some young teachers in interpersonal communication but also contribute to the completion of their scientific research tasks.

#### 6.2.3 Improving research environment

On the one hand, there are currently too many cumbersome procedures in school work, and the efficiency of handling affairs among organizational structures is low. Young teachers should devote their time and energy to teaching and scientific research tasks rather than running around among different organizational departments. When a person’s energy is divided by other matters, it is very difficult for them to concentrate on completing another task.

On the other hand, schools should establish specialized stress relief institutions to mediate the possible stress experienced by young teachers. They can also organize various interesting activities to enrich the daily lives of college teachers. By creating a favorable scientific research environment for young teachers in colleges and universities, the occupational pressure of young teachers can be alleviated (Yao and Zhang, 2021).

#### 6.2.4 Developing coping strategies & optimism

Young teachers in colleges and universities should have a correct understanding of the formulation of policies. Even though the current policy environment sets higher requirements for them, the policies are formulated based on certain grounds and not out of thin air. This indicates that the previous policies are no longer suitable for the current development, which is why new policies are put forward. Young teachers in colleges and universities should properly deal with pressure. When they are under pressure, they can appropriately slow down their pace of progress and relieve the pressure through means such as listening to music, playing ball games, and talking to others. Young teachers in colleges and universities should have full confidence in themselves. By maintaining an optimistic mindset and correctly facing challenges, they can effectively relieve the pressure.

## Data Availability

The raw data supporting the conclusions of this article will be made available by the authors, without undue reservation.
